# Contribution of the cold shock protein CspA to virulence in *Xanthomonas oryzae* pv. *oryzae*

**DOI:** 10.1111/mpp.12763

**Published:** 2018-11-16

**Authors:** Liming Wu, Liumin Ma, Xi Li, Ziyang Huang, Xuewen Gao

**Affiliations:** ^1^ College of Plant Protection Nanjing Agricultural University, Key Laboratory of Monitoring and Management of Crop Disease and Pest Insects, Ministry of Education Nanjing 210095 China

**Keywords:** ChIP, cold shock protein, transcriptome, virulence, *Xanthomonas*

## Abstract

*Xanthomonas oryzae* pv. *oryzae* (*Xoo*) causes a damaging bacterial leaf blight disease in rice. Cold shock proteins (Csps) are highly conserved nucleic acid‐binding proteins present in various bacterial genera, but relatively little is known about their functions in *Xanthomonas*. Herein, we identified four Csps (CspA–CspD) in the *Xoo* PXO99^A^ strain. Deletion of *cspA* decreased cold adaptation and a few known pathogenic factors, including bacterial pathogenicity, biofilm formation and polysaccharide production. Furthermore, we performed transcriptomic and chromosome immunoprecipitation (ChIP) experiments to identify direct targets of CspA and to determine its DNA‐binding sequence. Integrative data analysis revealed that CspA directly regulates two genes, *PXO_RS11830 *and *PXO_RS01060*, by binding to a conserved CCAAT sequence in the promoter region. We generated single‐deletion mutants of each gene and the results indicate that both are responsible for *Xanthomonas *pathogenicity. In addition, quantitative real‐time polymerase chain reaction and western blotting showed that CspA suppressed the expression of its direct targets. In summary, our study clarifies the characteristics of Csps in *Xanthomonas* and greatly advances our understanding of the mechanisms underlying the contribution of CspA to bacterial virulence.

## Introduction

Bacteria belonging to the genus *Xanthomonas* are Gram‐negative with a single polar flagellum and include many important plant pathogens affecting crops worldwide (Leyns *et al*., 1984). One member of this genus, *Xanthomonas oryzae* pv. *oryzae *(*Xoo*), is the causative agent of bacterial leaf blight that damages rice (Swings *et al*., 1990). This disease causes severe crop and economic losses, predominantly in tropical Asian countries (Ferluga *et al*., 2007). *Xoo* infects rice leaves primarily through hydathodes, which are natural openings situated at the tips and margins of rice leaves, as well as wounds at the leaf tips, broken trichomes, leaf margins and wounds in the leaves or roots, and then multiplies in intercellular spaces and enters xylem vessels, where it causes lesions (Nguyen *et al*., 2016). This bacterium has been defined as one of the top 10 plant‐pathogenic bacteria (Mansfield *et al*., 2012).

Successful infection and bacterial multiplication in host tissues generally depend on a wide range of virulence factors, including, but not limited to, extracellular polysaccharides (EPSs), lipopolysaccharides (LPSs), adhesins, biofilms and the type III secretion system (Büttner and Bonas, 2010). Xanthan, a characteristic EPS directed by several genetic loci, including the *gum* gene cluster (*gumB*–*gumM*), can lead to the appearance of mucoid bacterial colonies and cause wilting of host plants by blocking water flow in xylem vessels (Chan and Goodwin, 1999). LPSs are major components of the bacterial outer membrane that protects cells from the potentially hostile external environment (Meyer *et al*., 2001). Adhesins are required for bacterial attachment to the leaf surface and contribute to the bacterial colonization of xylem vessels (Das *et al*., 2009). Biofilm formation presumably provides protection against antibiotics and host defence responses, and may contribute to bacterial epiphytic survival before colonization of the plant intercellular space or the attachment of vascular bacteria to xylem vessels (Stoodley *et al*., 2002).

Bacterial cold shock proteins (Csps) are highly conserved, small (~7.4 kDa) proteins structurally related to nucleic acid‐binding proteins, and have been identified in a wide variety of Gram‐negative and Gram‐positive bacteria (Ermolenko and Makhatadze, 2002). In *Escherichia coli* and *Bacillus subtilis*, nine (CspA–CspI) and three (CspB–CspD) Csps have been characterized (Graumann *et al*., 1997; Jones *et al*., 1996). CspA (*E. coli*) and CspB (*B. subtilis*) are well‐studied DNA‐binding proteins with similar five‐stranded β‐barrel structures and moderately well‐conserved ribonucleoprotein 1 (RNP1; K/R‐G‐F/Y‐G/A‐F‐V/I‐X‐F/Y) and ribonucleoprotein 2 (RNP2; L/I‐F/Y‐V/I‐G/K‐N/G‐L) motifs (Graumann and Marahiel, 1996). Owing to significant sequence homology between Csps and the nucleic acid‐binding domains of eukaryotic gene‐regulatory Y‐box factors that recognize the ATTGG motif, as well as the complementary CCAAT Y‐box core sequence, Csps not only destabilize RNA secondary structure by acting as RNA chaperones, but also bind mRNA and thereby regulate ribosomal translation, mRNA decay and termination of transcription (Ermolenko and Makhatadze, 2002).

Although bacterial Csps are mainly induced after a rapid temperature downshift to regulate adaptation to cold stress, they are present under normal conditions to influence other biological functions. Schärer *et al*. (2013) and Eshwar *et al*. (2017) reported that Csps regulate the production of the pore‐forming cytolysin listeriolysin, haemolysis phenotypes, cell aggregation and flagella‐based motility in *Listeria monocytogenes*. Wang *et al*. (2014) showed that CspA plays an important role in stress adaptation and virulence in *Brucella melitensis*. Burbank and Stenger (2016) suggested a significant function of *Xylella fastidiosa* Csps on stress tolerance and pathogenicity in grapevine. However, the molecular mechanisms underlying the regulation of these functions remain unknown.

In this study, we identified Csps in *Xoo* PXO99^A^ by *in silico* analysis, and Csp‐encoding gene deletion mutants were constructed to investigate their effects on growth and virulence in *Xanthomonas*. Furthermore, we employed transcriptomic and chromatin immunoprecipitation (ChIP) analyses to explore the direct targets of CspA and to determine its binding sequence. The predicted CspA binding sequence and its interaction with CspA were confirmed using electrophoretic mobility shift assay (EMSA). Our results indicate that the CspA protein regulates optimum virulence in rice by direct regulation of the pathogenicity‐related genes *PXO_RS11830* and *PXO_RS01060* which encode a chemotaxis protein and glucan biosynthesis protein D. These findings shed light on the characteristics and mechanisms underlying the regulation of virulence by Csps in *Xanthomonas*.

## Results

### Identification of Csps in the genome of *Xoo* PXO99^A^

**Figure 1:**
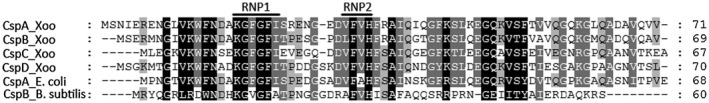
Multiple sequence alignment of cold shock proteins (Csps) from *Xanthomonas oryzae* pv. *oryzae* (*Xoo*) PXO99^A^, CspA from *Escherichia coli* and CspB from *Bacillus subtilis*. The canonical nucleic acid‐binding sequence motifs RNP1 and RNP2 are underlined. The numbers on the right represent the amount of amino acids. GenBank accession numbers for the listed species are as follows: CspA (*Xoo*), WP_011258294.1; CspB (*Xoo*), WP_011408744.1; CspC (*Xoo*), WP_011258799.1; CspD (*Xoo*), WP_003488188.1; CspA (*E. coli*), PXO_P0A9X9; CspB (*B. subtilis*), AAB01346.

Csps have been intensively characterized in *E. coli* and *B. subtilis*, and these are considered as models for bacterial Csp proteins (Graumann *et al*., 1997; Jones *et al*., 1996). To examine whether *Xoo* PXO99^A^ possesses Csps, we used *E. coli *CspA (P0A9X9) and *B. subtilis* CspB (AAB01346) as query sequences to perform a local BLASTP search of the genome of *Xoo* strain PXO99^A^. *In silico* analysis indicated that *Xoo* PXO99^A ^harbours four Csps that we renamed CspA–CspD; the corresponding genes were named *cspA*–*cspD*. It is apparent from Fig. 1 that CspA–CspD proteins share a remarkable degree of sequence identity with CspA (*E. coli*) and CspB (*B. subtilis*). For CspA, the identity was more than 60%. In addition, similarity was also found amongst CspA–CspD proteins. Importantly, the RNP1 and RNP2 canonical nucleic acid‐binding sequence motifs involved in the regulation of transcription and translation were identified (Landsman, 1992).

### CspA contributes to low‐temperature cellular growth and virulence against rice

**Figure 2:**
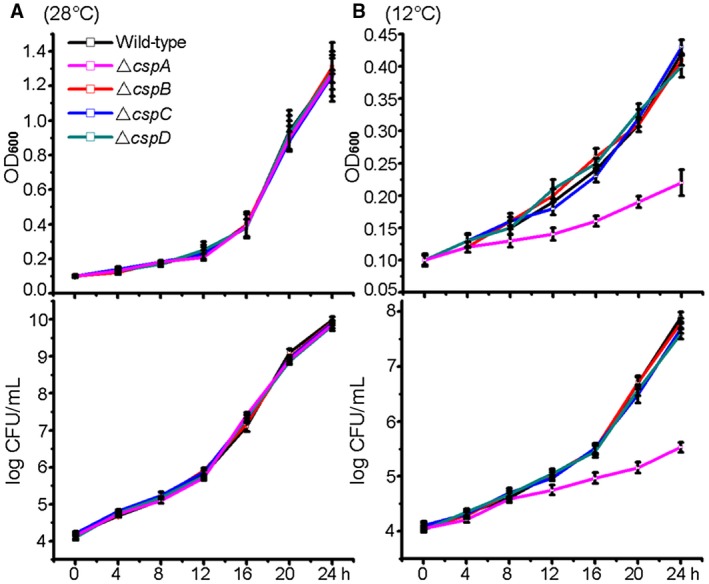
Growth at 28 °C (A) and 12 °C (B) of *Xanthomonas oryzae* pv. *oryzae* (*Xoo*) and the *ΔcspA*, *ΔcspB*, *ΔcspC* and *ΔcspD* mutants in nutrient broth (NB). Changes in cell density (optical density at 600 nm, OD_600_) and the number of bacterial cells [log colony‐forming units (CFU)/mL] were evaluated. Independent cultures were grown in triplicate and values represent the means ± standard deviation (*n* = 3). [Colour figure can be viewed at wileyonlinelibrary.com]

To study the role of Csps in *Xoo* PXO99^A^ cold adaptation, mutant strains were constructed for each *csp *gene by in‐frame deletion, without insertion of any antibiotic resistance cassette to avoid polar effects on surrounding genes. Growth at 28 °C in nutrient broth (NB) of the resulting *ΔcspA*, *ΔcspB*, *ΔcspC* and *ΔcspD* strains was similar to that of the wild‐type (WT) strain, and bacterial counts were in accordance with optical density at 600 nm (OD_600_) values (Fig. 2A). At 12 °C, growth of the *cspA *mutant was clearly impaired compared with WT cells, whereas deletion of *cspB*, *cspC* or *cspD *alone caused no such cold‐specific growth defect. The CFU value of the *ΔcspA* mutant decreased dramatically, whereas the other mutants showed no significant differences compared with WT cells (Fig. 2B).

**Figure 3:**
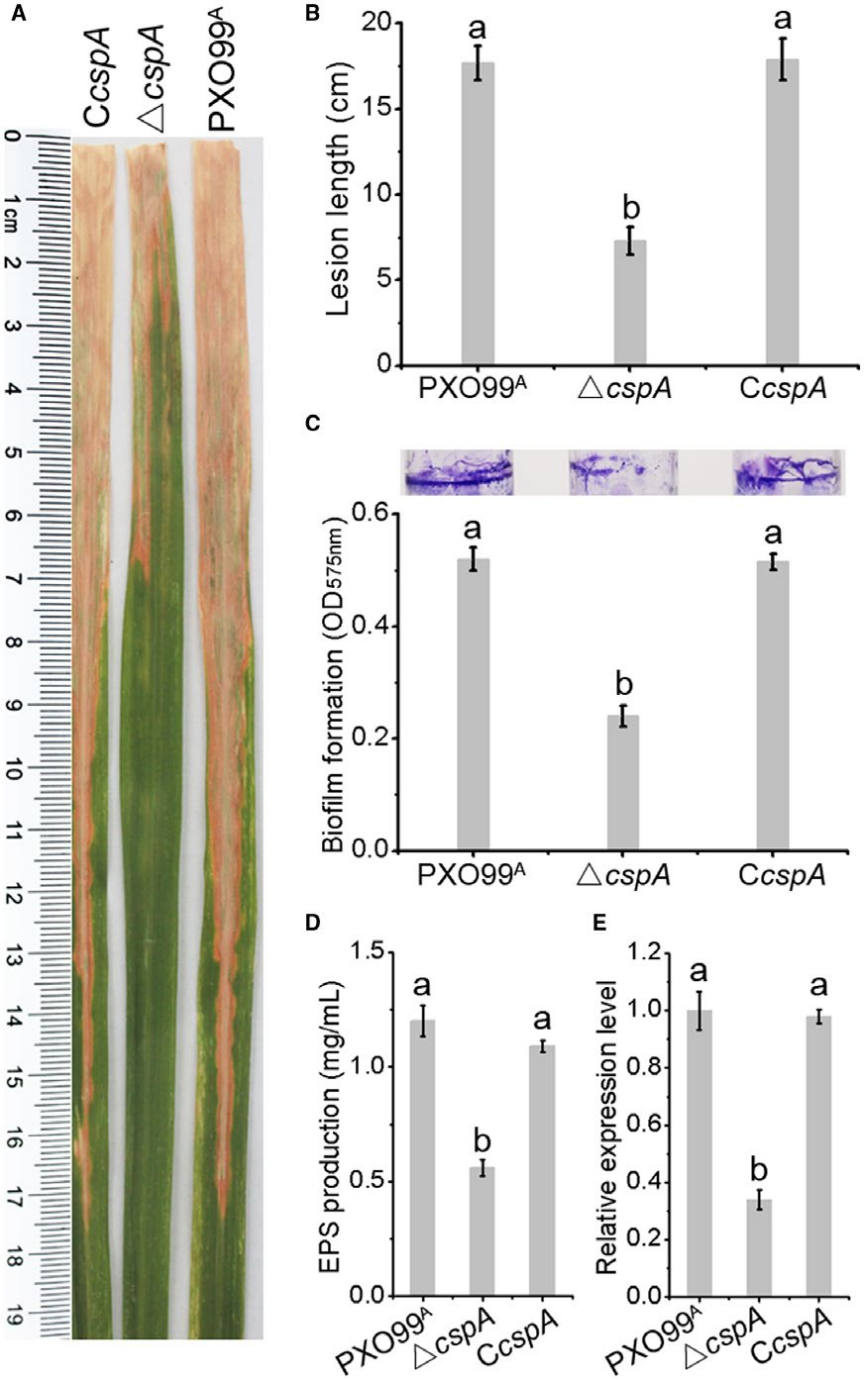
The *Xanthomonas oryzae* pv. *oryzae* (*Xoo*) *ΔcspA* mutant strain displays impaired virulence against rice and diminished biofilm formation and extracellular polysaccharide (EPS) production. (A) Representative results of lesion length symptoms caused by *Xoo* strains in susceptible rice leaves. (B) Calculated lesion lengths on the leaves of susceptible adult rice plants. (C) Qualitative and quantitative analysis of biofilm formation by PXO99^A^, *ΔcspA* (deletion) and C*cspA* (complemented) strains on glass tubes. (D) EPS production in wild‐type (WT) and mutant *Xoo* strains. (E) Transcription of the *gumK* gene involved in EPS biosynthesis in different strains. Data are means ± standard deviation (SD). Letters indicate a significant difference (*P* < 0.05). [Colour figure can be viewed at wileyonlinelibrary.com]

To investigate whether Csps contribute to *Xoo* virulence, mutants were used to inoculate the susceptible host rice. Figure 3A,B reveals that, compared with WT cells, the *ΔcspA* mutant exhibited a twofold decrease in lesion length in rice leaves at 14 days post‐inoculation (dpi). Interestingly, co‐expression of full‐length WT CspA in the *ΔcspA* mutant using the broad‐host‐range vector pHM1 was able to restore disease symptoms and lesion length. Under similar test conditions, the lengths of lesions caused by the three mutants were similar to those caused by the WT strain (Fig. S1, see Supporting Information). These results indicate that CspA is important for cellular growth at low temperature and for virulence against rice.

### Role of CspA in virulence‐associated factors

Biofilm formation and EPS production are important virulence‐associated factors in *Xanthomonas* (Büttner and Bonas, 2010). As mutation of the *cspA* gene decreased virulence in *Xoo*, it was of interest to determine whether the contribution of *cspA* to virulence was dependent on its ability to regulate these known virulence‐associated factors. The results showed that inactivation of *cspA* caused a twofold reduction in biofilm formation following staining with crystal violet, and the complemented strain exhibited levels comparable with the WT strain (Fig. 3C). In the EPS assay, the *ΔcspA* mutant displayed significantly impaired EPS production and the complemented strain restored production to WT levels (Fig. 3D). Transcription of *gumK*, a member of the *gum* operon responsible for EPS biosynthesis, was markedly decreased in the *ΔcspA* mutant (Fig. 3E), consistent with the result of EPS production. These results reveal that the CspA protein is important for biofilm formation and EPS production.

### Identification of genes and targets regulated by CspA using transcriptomic and ChIP analyses

**Figure 4:**
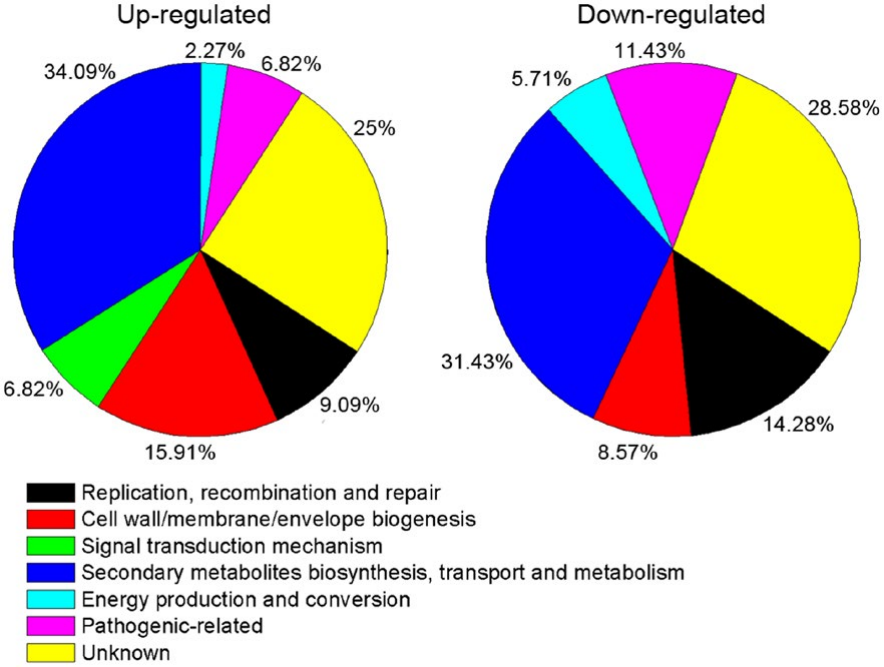
Functional categories of genes exhibiting altered transcription in the *ΔcspA* mutant compared with the wild‐type (WT) *Xanthomonas oryzae* pv. *oryzae* (*Xoo*) strain. Fifty‐eight genes with known functions were classified, whereas 21 were of unknown function. [Colour figure can be viewed at wileyonlinelibrary.com]

To experimentally identify CspA‐regulated genes, we first performed RNA sequencing (RNA‐seq) analysis to obtain the regulatory profile of CspA by comparison of WT *Xoo* PXO99^A^ and *ΔcspA* mutant cells. Inactivation of *cspA* significantly altered the expression of 79 genes, 35 of which were down‐regulated and 44 of which were up‐regulated (Table S3, see Supporting Information). Deletion of *cspA* led to altered expression of a set of genes associated with a range of biological functions, including bacterial pathogenicity, secondary metabolite secretion and signal transduction (Fig. 4).

CspA is a DNA‐binding protein possessing nucleic acid‐binding domains. To identify the consensus CspA binding sequence, ChIP and high‐throughput sequencing (ChIP‐seq) were performed to screen for CspA‐binding DNA sequences at a genome‐wide scale. A recombinant *Xoo* PXO99^A^ strain containing the pHM1::*cspA*‐his_6_ vector was constructed and a monoclonal anti‐His_6_ antibody was used to probe the CspA‐His_6_ protein bound to its DNA target. Following high‐throughput sequencing, peak calling revealed that CspA binds at 87 genomic sites, including the putative promoter regions of 19 genes for which the functional classifications are summarized in Table S4 (see Supporting Information).

**Figure 5:**
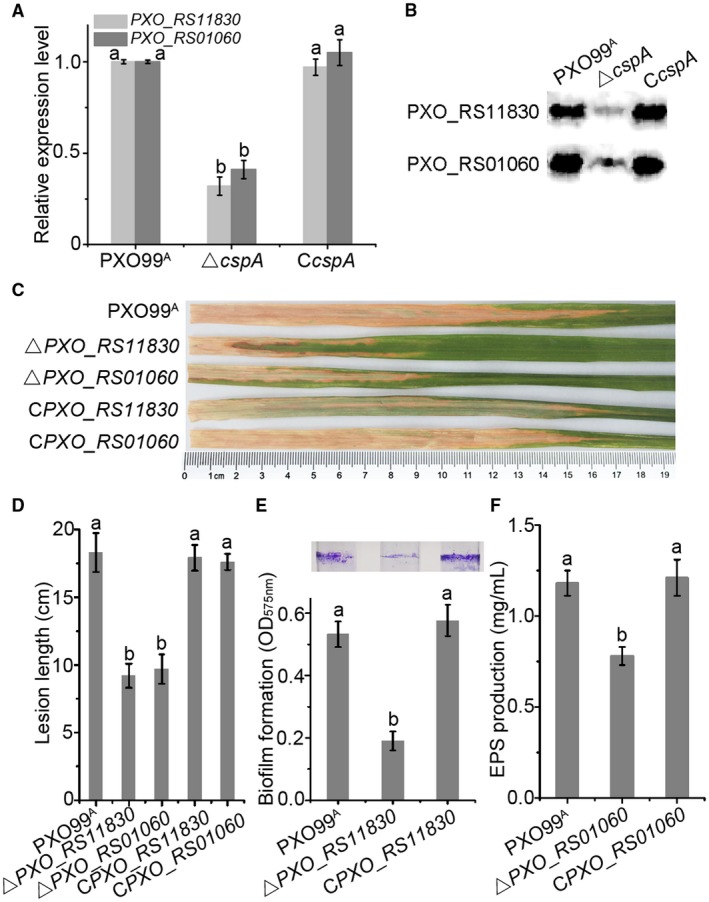
Genes *PXO_RS11830* and *PXO_RS01060* are important for *Xanthomonas oryzae* pv. *oryzae* (*Xoo*) pathogenicity against rice. (A) Transcriptional levels of *PXO_RS11830* and *PXO_RS01060* genes monitored by quantitative real‐time polymerase chain reaction (PCR). (B) Levels of proteins encoded by *PXO_RS11830* and *PXO_RS01060* detected by western blotting. (C) Lesion length symptoms in *ΔPXO_RS11830*, *ΔPXO_RS01060* and complemented strains. (D) Calculated lesion lengths on leaves. (E) The *ΔPXO_RS11830* deletion strain displays impaired biofilm formation. (F) The *ΔPXO_RS01060* strain exhibits defective extracellular polysaccharide (EPS) production. [Colour figure can be viewed at wileyonlinelibrary.com]

Comprehensive analysis of the transcriptomic and ChiP results revealed two genes, *PXO_RS11830 *encoding a chemotaxis protein and *PXO_RS01060* encoding glucan biosynthesis protein D (GbpD), for which CspA binds the promoter and significantly down‐regulates expression. To validate the transcriptomic results, the expression levels of these two genes were measured by quantitative reverse transcription‐polymerase chain reaction (qRT‐PCR) and western blot analysis. The results in Fig. 5A,B indicate that transcription of *PXO_RS11830* and *PXO_RS01060* was markedly down‐regulated and the protein level was also markedly lower in the *ΔcspA* mutant compared with WT cells, consistent with the transcriptomic data. Simultaneously, we knocked out *PXO_RS11830 *and *PXO_RS01060 *in the *Xoo* PXO99^A^ strain. As shown in Fig. 5C–F, the *PXO_RS11830 *mutation impaired biofilm formation and virulence, whereas the *PXO_RS01060* mutant also displayed weaker EPS production and virulence than the WT strain, and complemented strains restored levels to those of WT cells.

### Identification and verification of the conserved CspA binding sequence

**Figure 6:**
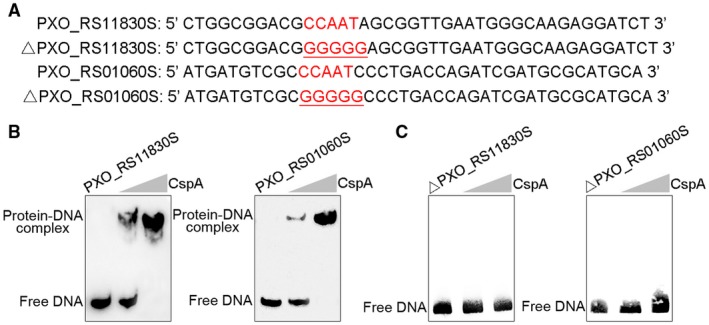
The CspA protein regulates *PXO_RS11830* and *PXO_RS01060* genes and binds to the conserved CCAAT sequence within the promoter region. (A) Sequences of probes used in the electrophoretic mobility shift assay (EMSA) experiments. Conserved nucleotides CCAAT are coloured red and mutations are underlined. (B) CspA binds to motifs located within the promoter regions of *PXO_RS11830* and *PXO_RS01060*. EMSA experiments were conducted in the presence of purified 6His‐CspA and the corresponding probes. (C) Influences of mutated nucleotides on CspA–DNA interaction. [Colour figure can be viewed at wileyonlinelibrary.com]

On the basis of the above findings, the CCAAT consensus CspA‐binding DNA sequence was predicted from the putative promoter regions of CspA‐regulated genes, including *PXO_RS11830* and *PXO_RS01060*. Previous reports have demonstrated that Csps bind with high affinity to the CCAAT sequence (Ermolenko and Makhatadze, 2002; Graumann and Marahiel, 1994). To verify whether CspA binds to the CCAAT sequence *in vitro*, purified 6His‐CspA and 40‐bp oligonucleotide probes containing the CspA binding sequence in the promoter regions of *PXO_RS11830 *and *PXO_RS01060 *(Fig. 6A) were selected to interrogate DNA–protein interactions by EMSA. Motility shifts were observed for DNA probes on addition of purified 6His‐CspA protein (Fig. 6B) and mutation of the CCAAT sequence to GGGGG abolished this motility shift (Fig. 6C), suggesting that these conserved nucleotides are critical for CspA binding and play an important role in protein–DNA interactions.

## Discussion

Bacterial Csps are highly conserved small multifunctional nucleic acid‐binding proteins that mediate a wide range of physiological functions, including stress resistance and virulence‐associated responses, by modulating transcription, translation and mRNA stability (Eshwar *et al*., 2017; Schärer *et al*., 2013). Csps have been identified as major determinants of pathogenicity in various disease‐causing bacteria, including *Enterococcus faecalis*, *Staphylococcus aureus*, *Salmonella enterica*, *Brucella melitensis* and *Listeria monocytogenes* (Eshwar *et al*., 2017; Michaux *et al*., 2012, 2017; Sahukhal and Elasri, 2014; Wang *et al*., 2014). Despite extensive research during the last decade, the molecular mechanism underlying the association of Csps and virulence has not been fully elucidated. In this study, we showed that CspA of *Xoo* is not only involved in the cold adaptation response, but also facilitates virulence against rice. Importantly, CspA can bind to pathogenicity‐related genes encoding a chemotaxis protein and glucan biosynthesis protein D, thereby enhancing virulence.

In *Xoo* strain PXO99^A^, there are four Csps (CspA–CspD) (Fig. 1). Single‐gene deletions of *cspB*, *cspC* and *cspD* did not result in cold‐specific growth defective phenotypes, but deletion of *cspA *resulted in a marked reduction in growth rate at 12 °C (Fig. 2). These results indicate a hierarchy of importance to cellular growth at low temperatures within the Csp family, with CspA conferring the most important function. Similar results have been reported by Graumann *et al*. (1997) showing that CspB is essential for efficient adaptation to low temperatures in *B. subtilis*.

An important finding of this work was that the *ΔcspA* strain was less virulent against rice than the WT strain, whereas *cspB*–*cspD* mutations had no significant impact (Figs 3A, S1). Consistent with the involvement of CspA in *Xanthomonas *virulence modulation, we also showed that the *ΔcspA* mutant displayed significantly impaired biofilm formation (Fig. 3C). In addition, deletion of *cspA *lowered the amount of the virulence factor EPS (Fig. 3D), in accordance with the observed down‐regulation of the *gumK* gene (Fig. 3E) encoding a membrane‐associated β‐glucuronosyltransferase which is responsible for EPS biosynthesis. Our current results are similar to reports of other bacteria linking Csps to the regulation of virulence. Previously, Schärer *et al*. (2013) have reported a reduction in the virulence protein listerolysin O in a CspB deletion mutant of *L. monocytogenes*, and a *Vibrio cholerae* strain lacking *cspV* displayed defective biofilm formation and type VI secretion (Townsley *et al*., 2016).

Csps belong to a large family of structurally related nucleic acid‐binding proteins that bind RNA and DNA (Ermolenko and Makhatadze, 2002). Based on homology between Csps and Y‐box proteins, which are known to play a role in transcription, they were predicted to bind the promoter region of various genes to modify their transcription (Horn *et al*., 2007). Hofweber *et al*. (2005) revealed that *B. subtilis* CspB has an inhibitory effect on the gene encoding chloramphenicol acetyltransferase. To elucidate the molecular mechanism of CspA in the regulation of virulence, by employing transcriptomic analyses, we identified 79 genes regulated by CspA, 35 of which were down‐regulated and 44 of which were up‐regulated (Fig. 4; Table S4). Although transcriptomic analysis provides a comprehensive view of the genome‐wide gene expression profile of CspA, it cannot distinguish between direct targets and indirectly regulated genes. By contrast, ChIP‐seq is a widely applied tool for the identification of DNA‐binding sites and targets of regulators (Johnson *et al*., 2007). Among the 19 direct targets of CspA identified by ChIP‐seq (Table S3), only two pathogenic genes (*PXO_RS11830 *and *PXO_RS01060*) were identified by transcriptomic analysis. The conserved CCAAT binding sequence of CspA was identified and confirmed in the promoter regions of both *PXO_RS11830 *and *PXO_RS01060 *genes. We further defined and experimentally validated the CspA binding sequence by EMSA. Mutation of these conserved nucleotides effectively destroyed protein–probe binding (Fig. 6). These results are in accordance with the conclusion of Graumann and Marahiel (1994) that CspB binds with strong affinity to cis‐elements containing ATTGG or CCAAT sequences, and exhibits low or no affinity to altered core sequences.

Gene *PXO_RS11830 *encodes a chemotaxis protein, and the product of the *PXO_RS01060 *gene is glucan biosynthesis protein D. Shen *et al*. (2001) suggested that chemotaxis plays an important role in *Xoo* pathogenicity, and glucan biosynthesis mutants of *X. axonopodis* and *X. campestris* display weakened virulence (Astua‐Monge *et al*., 2005; Minsavage *et al*., 2004). Similarly, in the PXO99^A^ strain, mutation of *PXO_RS11830 *and *PXO_RS01060 *genes resulted in reduced virulence and decreased pathogenicity factor production (Fig. 3). Meanwhile, expression of these two genes was markedly repressed in the absence of the *cspA* gene, at both mRNA and protein levels, as demonstrated by qRT‐PCR and western blotting (Fig. 3A,B).

In conclusion, we identified four Csps in *Xoo *PXO99^A^ and found that CspA is important for cold adaptation and virulence against rice. Combining transcriptomic and ChIP data, we revealed that CspA binds the conserved CCAAT sequence located within the promoter regions of pathogenicity‐related genes *PXO_RS11830 *and *PXO_RS01060*. These findings provide new insights into the role of Csps in bacteria and help us to comprehensively understand their molecular mechanisms in the regulation of pathogenicity.

## Experimental Procedures

### Bacterial strains and culture conditions

All bacterial strains and plasmids used in this work are listed in Table S1 (see Supporting Information). *Xanthomonas* strains were grown at 28 °C in NB medium (beef extract, 3 g/L; yeast extract, 1 g/L; polypeptone, 5 g/L; sucrose, 10 g/L) or on nutrient agar (NA). *Escherichia coli* strains were cultivated at 37 °C in Luria–Bertani (LB) broth or on LB agar plates. When required, antibiotics were added at the following final concentrations: ampicillin (Amp), 100 μg/mL; kanamycin (Km), 50 μg/mL; spectinomycin (Sp), 100 μg/mL.

### Generation of mutants and complemented strains

In‐frame deletion mutants and complemented strains were generated as described by Qian *et al*. (2013). In brief, two flanking regions were generated and ligated into the pK18*mobsacB* plasmid. The resulting recombinant vectors were introduced into WT PXO99^A^ cells via electroporation. Transformants were selected on NA plates without sucrose, but containing 50 μg/mL Km. Positive colonies were then plated onto NA plates containing 10% (w/v) sucrose to select for resolution of the construct by a second cross‐over event. The resulting in‐frame deletion mutants were confirmed by PCR analysis. For complementation, complete target genes were cloned into the broad‐host‐range vector pHM1 and the resulting plasmids were transferred into the corresponding mutant via electroporation to generate complemented strains.

### Cold adaptation assay

To study the effect of cold shock on survival in *Xanthomonas*, cultures were grown to mid‐logarithmic phase, with an OD_600_ value of 0.5 in NB medium. Aliquots of 5 mL were transferred into fresh NB medium to a final OD_600_ of ~0.1 and incubated at 28 °C with shaking at 200 rpm or at 12 °C for 24 h. Growth curves were generated by the measurement of OD_600_ and colony‐forming unit (CFU) values every 4 h.

### Rice plant virulence assays

Briefly, susceptible rice cultivar IR24 plants were grown in a growth chamber under a cycle of 12 h of light at 28 °C and 12 h of dark at 25 °C with ~70% relative humidity. Leaf tips from 40‐day‐old plants were removed with sterile scissors, dipped in bacterial suspensions at OD_600_ ~ 0.5, and lesion lengths and representative images were recorded after 2 weeks.

### Biofilm formation assay and quantification of EPS production

Biofilm formation assays were performed as described previously (Wang *et al*., 2018). Bacterial cells were cultured in NB medium to OD_600_ ~ 1.0 and 4 mL of cell suspensions were transferred to sterilized tubes and incubated at 28 °C for 7 days without shaking. Cultures were discarded and tubes were rinsed with water. Biofilm formation was visualized by staining with 0.1% crystal violet, and the stained biofilm was dissolved in methanol–acetic acid–water (4 : 1 : 5, v/v/v) and quantified by measurement of the absorbance at a wavelength of 575 nm using a spectrophotometer.

EPS production was measured as described by Qian *et al*. (2013). Briefly, bacterial strains were grown in NB broth containing 4% glucose at 28 °C with shaking at 200 rpm for 5 days. Cells were removed by centrifugation at 5000 ***g*** for 20 min and the culture supernatant was mixed with three volumes of ethanol and kept at 4 °C for 30 min. To determine the dry weight of EPS, the precipitated EPS was centrifuged and dried at 80 °C to constant weight. Three independent replicates were used for each strain.

### Total RNA extraction and transcriptomic analysis

A single colony of PXO99^A^ WT and *ΔcspA* mutant cells was inoculated into NB broth and incubated at 28 °C with shaking at 200 rpm to exponential growth phase (OD_600_ ~ 1.0). Total RNA was extracted using a Bacterial RNA Kit (Omega Bio‐Tek, Doraville, GA, USA) according to the manufacturer’s instructions. RNA quantity and quality were assessed using an RNA Nano 6000 Assay Kit (Agilent Technologies, Santa Clara, CA, USA). For cDNA library construction and deep sequencing, RNA samples were prepared using a TruSeq RNA Sample Preparation Kit (Illumina Inc., San Diego, CA, USA), and libraries were sequenced using the Illumina HiSeq2000 platform in accordance with the manufacturer’s protocol (Illumina Inc., San Diego, CA, USA).

Transcriptomic data were analysed as described previously (Lee *et al*., 2017). Briefly, gene ontology (GO) and Kyoto Encyclopedia of Genes and Genomes (KEGG) enrichment analysis were carried out for biological process and molecular function categories. The analysis of differentially expressed genes (DEGs) was based on the DESeq package described by Anders and Huber (2010), with the false discovery rate (FDR) used to determine DEGs. In this study, transcripts were designated as significant DEGs when they exhibited at least a twofold change in expression level (Xie *et al*., 2015).

### ChIP assay

ChIP assays were performed according to the procedures described by Zhang *et al*. (2012). Briefly, *Xoo* strain PXO99^A^ harbouring the pHM1‐cspA‐his plasmid was grown in 20 mL of NB medium to OD_600_ ~ 1.0. Proteins and chromatin DNA were cross‐linked by the addition of formaldehyde to a final concentration of 1% for 10 min and the cross‐linking reaction was stopped by the addition of glycine. The assay was performed using a ChIP assay kit (Millipore, Billerica, MA, USA) following the manufacturer’s instructions. A small aliquot of untreated sonicated chromatin was reverse cross‐linked and used as the total input DNA control. The resulting DNA was purified using a PCR purification kit (Qiagen, Hilden, Germany). Sequencing was performed using an Illumina HiSeq 2000 system (Illumina Inc.). Cleaned reads were aligned to the genomic sequence of *Xoo* PXO99^A^.

### Protein expression, purification and antibody production

The pET‐30a‐c(+) expression vector (Novagen, Madison, Wisconsin, USA) was used to express N‐terminal hexahistidine (His_6_)‐tagged proteins. Recombinant vectors were transformed into the *E. coli* BL21 (DE3) strain, and protein expression and purification protocols were performed as described by Zang *et al*. (2015). In brief, *E. coli* strains were grown to OD_600_ ~ 0.6 and isopropyl‐β‐d‐thiogalactoside was then added to a final concentration of 0.2 mm and culturing was continued for 4–6 h at 28 °C. Cells were collected by centrifugation and treated with imidazole buffer [dissolved in phosphate‐buffered saline (PBS) containing 20 μg/mL phenylmethylsulfonylfluoride (PMSF)]. Crude protein supernatants were loaded onto an Ni‐NTA column (GE Healthcare Life Science, Piscataway, NJ, USA). Purified tagged proteins were dissolved in storage buffer [50 mm TRIS‐HCl, pH 8.0, 0.5 mm ethylenediaminetetraacetic acid (EDTA), 50 mm NaCl and 5% glycerol].

The purified recombinant chemotaxis protein and GbpD proteins were injected with a complete Freund adjuvant into New Zealand white rabbits. After three injections (one every 2 weeks), serum was collected and tested for anti‐chemotaxis protein and anti‐GbpD antibodies.

### EMSA and western blotting

Probe oligonucleotides were synthesized by Genscript Biotechnology Corporation (Nanjing, China). DNA labelling was performed using a Pierce Biotin 3′ End DNA Labelling Kit (Beyotime, Shanghai, China). Samples were size fractionated using a 4% non‐denaturing polyacrylamide gel run at 60 V for 2 h at 4 °C in 0.5 × TBE buffer (45 mm TRIS‐borate and 1 mm EDTA). The gel was transblotted onto a Zeta‐Probe Membrane (Bio‐Rad, Hercules, CA, USA) using a Mini Trans‐Blot Cell (Bio‐Rad) at 380 mA for 1 h. The membrane was then treated with a Chemiluminescent EMSA Kit (Beyotime) and detected by a ChemiDo MP System (Bio‐Rad).

The levels of chemotaxis protein and GbpD were measured by western blotting. Soluble proteins were harvested from *Xoo* PXO99^A^ WT and *ΔcspA* mutant cells, further separated by sodium dodecylsulfate‐polyacrylamide gel electrophoresis (SDS‐PAGE) and immobilized onto a polyvinylidene difluoride (PVDF) membrane using a semi‐dry blot machine (Bio‐Rad). Membranes were probed with antibodies, followed by detection with a horseradish peroxidase (HRP)‐conjugated anti‐rabbit secondary antibody (Abmart, Shanghai, China).

### Quantitative real‐time PCR analysis

First‐strand cDNA was synthesized with random hexamer primers using reverse transcriptase (TaKaRa Bio Inc., China) and the resulting cDNA was used as template for subsequent PCR amplification. qRT‐PCR was conducted with SYBR Premix Ex Taq (TaKaRa Bio Inc.) using a 7500 Fast Real‐Time PCR Detection System. The *16S rRNA* gene was used as an internal reference for normalization. All primers used are listed in Table S2 (see Supporting Information).

### Statistical analysis

Each experiment was conducted at least in triplicate. Data were statistically evaluated using analysis of variance, followed by Fisher’s least‐significant difference tests (*P* ≤ 0.05) using SPSS ver. 16.0 software (Chicago, IL, USA).

## Supporting information


**Fig. S1** Mutants (ΔcspB, ΔcspC and ΔcspD) showed a lesion length on rice leaves similar to that of the wild‐type.

**Table S1** Bacterial strains and plasmids used in this study.

**Table S2** Primers used in this study.

**Table S3** The genes differentially expressed in the ΔcspA mutant compared with the wild‐type.

**Table S4** Nineteen putative CspA DNA‐binding genes identified by chromosome immunoprecipitation and high‐throughput sequencing (ChIP‐seq) analysis.
