# Supplementary material for: Contribution of the cold shock protein CspA to virulence in Xanthomonas oryzae pv. oryzae
Source: Mol Plant Pathol. 2018 Nov 16;20(3):382–91. doi: 10.1111/mpp.12763 (PMC6637868; doi:10.1111/mpp.12763)
Supplement: Supplementary file 1 — Fig. S1 Mutants (ΔcspB, ΔcspC and ΔcspD) showed a lesion length on rice leaves similar to that of the wild‐type. [file MPP-20-382-s001.docx]

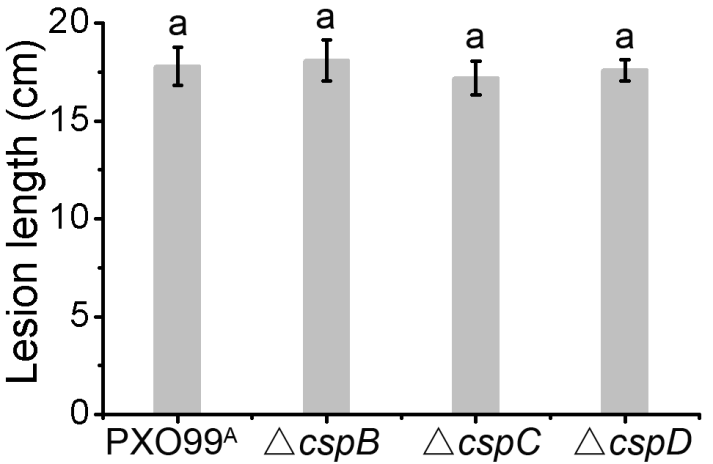


**Fig. S1 Mutants (*△cspB*, *△cspC* and *△cspD*) showed a lesion length on rice leaves similar to that of the wild-type.**
