# Supplementary material for: Contribution of the cold shock protein CspA to virulence in Xanthomonas oryzae pv. oryzae
Source: Mol Plant Pathol. 2018 Nov 16;20(3):382–91. doi: 10.1111/mpp.12763 (PMC6637868; doi:10.1111/mpp.12763)
Supplement: Supplementary file 2 — Table S1 Bacterial strains and plasmids used in this study. [file MPP-20-382-s002.docx]

**Table S1. Bacterial strains and plasmids used in this study**

| **Strains or plasmids** | **Relevant genotype, characteristics** | **Source or reference** |
| --- | --- | --- |
| **Strains** |  |  |
| *Xanthomonas oryzae* pv. *oryzae* |  |  |
| PXO99^A^ | Philippine race 6; wild-type strain (WT) | Salzberg et al., (2008) |
| *△cspA* | *cspA* in-frame deletion mutant of strain PXO99^A^ | This study |
| *△cspB* | *cspB* in-frame deletion mutant of strain PXO99^A^ | This study |
| *△cspC* | *cspC* in-frame deletion mutant of strain PXO99^A^ | This study |
| *△cspD* | *cspD* in-frame deletion mutant of strain PXO99^A^ | This study |
| C*cspA* | *△cspA* harbouring plasmid pHM1-*cspA* (complemented strain); Sp^R^ | This study |
| *△PXO_RS11830* | *PXO_RS11830* in-frame deletion mutant of strain PXO99^A^ | This study |
| *△PXO_RS01060* | *PXO_RS01060* in-frame deletion mutant of strain PXO99^A^ | This study |
| C*PXO_RS11830* | *△PXO_RS11830* harbouring plasmid pHM1- *PXO_RS11830* (complemented strain); Sp^R^ | This study |
| C*PXO_RS01060* | *△PXO_RS01060* harbouring plasmid pHM1- *PXO_RS01060* (complemented strain); Sp^R^ | This study |
| CspA-His | CspA-His_6_, WT strain containing a recombinant vector; pHM1::*cspA-his_6_*, Sp^R^ | This study |
| *Escherichia coli* |  |  |
| DH5α | *F ^–^,Φ80dlacZ ΔM12 minirecA1* | TaKaRa Bio Inc. |
| BL21(DE3) | *F ^–^, ompT, gal, dcm, lon, k(DE3)* | TaKaRa Bio Inc. |
| **Plasmids** |  |  |
| pK18*mobsacB* | Allelic exchange suicide vector, *sacB* oriT(RP4); Km^R^ | Zhao et al. (2012) |
| pHM1 | Broad-host-range *cos* IncW derivative of pRI40, Sp^R^ | Zhao et al. (2012) |
| pMD19-T simple | cloning vector; Amp^R^ | TaKaRa Bio Inc. |
| pET-30a-c(+) | Protein expression vector; Km^R^ | TaKaRa Bio Inc. |

Resistance marker: Amp^R^, ampicillin resistance; Km^R^, kanamycin resistance; Sp^R^, spectinomycin resistance.
