# Supplementary material for: Contribution of the cold shock protein CspA to virulence in Xanthomonas oryzae pv. oryzae
Source: Mol Plant Pathol. 2018 Nov 16;20(3):382–91. doi: 10.1111/mpp.12763 (PMC6637868; doi:10.1111/mpp.12763)
Supplement: Supplementary file 3 — Table S2 Primers used in this study. [file MPP-20-382-s003.docx]

**Table S2. Primers used in this study**

| **Name** | **Sequence (5´-3´)** |
| --- | --- |
| **Construct mutants** | |
| cspA1-F | CGGGATCCTGGGCAAGATGTAGTAAACGA (*Bam*H I) |
| cspA1-R | CCCAAGCTTCGCGGCTAATGAATCCAAA (*Hin*d III) |
| cspA2-F | CCCAAGCTTAGCCTCAAGGAAGGTCAGAAGG (*Hin*d III) |
| cspA2-R | GCTCTAGAGCAGCGTCGCCAGGAAATA (*Xba* I) |
| cspB1-F | CGGGATCCAAGCGGTTTCATCTGGTTATTT (*Bam*H I) |
| cspB1-R | CCCAAGCTTGAAGCCCTTGGCATCGTTA (*Hin*d III) |
| cspB2-F | CCCAAGCTTATGCAGGCTGACCAGGTGC (*Hin*d III) |
| cspB2-R | GCTCTAGAAGGCGATTTGCCGAGTGG (*Xba* I) |
| cspC1-F | CGGGATCCCGCCAACACCGACATCCT (*Bam*H I) |
| cspC1-R | CCCAAGCTTGAAGCCAACGCCTTTGTGA (*Hin*d III) |
| cspC2-F | CCCAAGCTTAGATCATCACCTATGCAATCGA (*Hin*d III) |
| cspC2-R | GCTCTAGATAACGCCGCAAACCCTGT (*Xba* I) |
| cspD1-F | CGGGATCCGCAGCAAGCCCATATTCCA (*Bam*H I) |
| cspD1-R | ACGCGTCGACCGTCAGTTATGACGTGACCCA (*Sal* I) |
| cspD2-F | ACGCGTCGACTGATCGCGGAGAAGTGCG (*Sal* I) |
| cspD2-R | GCTCTAGACCTGGAATATGCGTGTCTGG (*Xba* I) |
| CcspA-F | CCCAAGCTTATGTCCAACATCGAACGCGA (*Hin*d III) |
| CcspA-R | GGAATTCTCAGACCACCTGCACTGCGT (*Eco*R I) |
| PXO_RS118301-F | CGGGATCCATGTATTCACGTCCACTCAT (*Bam*H I) |
| PXO_RS118301-R | CCCAAGCTTGAAGCGGGCGACGTCGACGC (*Hin*d III) |
| PXO_RS118302-F | CCCAAGCTTTCCAAGGAATCGATCGCCAA (*Hin*d III) |
| PXO_RS118302-R | GCTCTAGATTAGAACAACTCGACCGTGC (*Xba* I) |
| PXO_RS010601-F | CGGGATCCATGCAACGACGCCACTTGCT (*Bam*H I) |
| PXO_RS010601-R | CCCAAGCTTTCGATCGCCAGCCCGCGCGC (*Hin*d III) |
| PXO_RS010602-F | CCCAAGCTTTGCAGTTGGTCGAGATTCCG (*Hin*d III) |
| PXO_RS010602-R | GCTCTAGATCAATTCTTGCGCTCGCTCGC (*Xba* I) |
| CPXO_RS11830-F | CCCAAGCTTATGTATTCACGTCCACTCAT (*Hin*d III) |
| CPXO_RS11830-R | GGAATTCTTAGAACAACTCGACCGTGC (*Eco*R I) |
| CPXO_RS01060-F | CCCAAGCTTATGCAACGACGCCACTTGCT (*Hin*d III) |
| CPXO_RS01060-R | GGAATTCTCAATTCTTGCGCTCGCTCGC (*Eco*R I) |
| **ChIP assay** | |
| CspAhis-F | GGGGTACCATGTCCAACATCGAACGCGA (*Kpn* I) |
| CspAhis-R | CGGAATTCTCAATGGTGATGGTGATGATGGACCACCTGCACTGCGTCGG (*Eco*R I) |
| **Quantitative real time-PCR analysis** | |
| 16S RNA-F | TGGCAACTAAGGACAAGGG |
| 16S RNA-R | AAGCGGTGGAGTATGTGG |
| gumK-F | AGAGACCCGGACCAGGTGTTA |
| gumK-R | TTGGTGAACGGGATCGGCAGCG |
| RTPXO_RS11830-F | CCAACACCGTCCATCACATC |
| RTPXO_RS11830-R | TGGAACGCTCGGACAGATT |
| RTPXO_RS01060-F | TTCCATCTTGGGCTGTATTTCC |
| RTPXO_RS01060-R | GATTGTCCGTACTGGCCTTCC |
| **Protein expression** | |
| PcspA-F | CGGGATCCAATGGTCTCGTGAAGTGGTT (*Bam*H I) |
| PcspA-R | CGGAATTCTCAGACCACCTGCACTGCGT (*Eco*R I) |
| PPXO_RS11830-F | CGGGATCCGCCGCTCCTTTGGCTCTGAC (*Bam*H I) |
| PPXO_RS11830-R | CCCAAGCTTTTAGAACAACTCGACCGTGCC (*Hin*d III) |
| PPXO_RS01060-F | CGGGATCCAATGCTGCCGCCGCATTGGC (*Bam*H I) |
| PPXO_RS01060-R | CCCAAGCTTTCAATTCTTGCGCTCGCTCGC (*Hin*d III) |

Restriction sites in primers are underlined.
