# Supplementary material for: Contribution of the cold shock protein CspA to virulence in Xanthomonas oryzae pv. oryzae
Source: Mol Plant Pathol. 2018 Nov 16;20(3):382–91. doi: 10.1111/mpp.12763 (PMC6637868; doi:10.1111/mpp.12763)
Supplement: Supplementary file 4 — Table S3 The genes differentially expressed in the ΔcspA mutant compared with the wild‐type. [file MPP-20-382-s004.docx]

**Table S3. The genes differentially expressed in △*cspA* mutant compared to the wild type.**

| **Genes** | **Annotation** | **Fold-change ratio** |
| --- | --- | --- |
| **Differentially expressed genes (79)** | | |
| **Up-regulated genes (44)** | | |
| Replication, recombination and repair (9.09%) | | |
| *PXO_RS05365* | Transposase | 2.5757 |
| *PXO_RS08460* | Chromosome partitioning protein ParA | 2.5897 |
| *PXO_RS27405* | Transposase | 2.6899 |
| *PXO_RS28095* | Putative transposase of IS4/5 family | 2.0169 |
| Cell wall/membrane/envelope biogenesis (15.91%) | | |
| *PXO_RS04270* | membrane protein | 2.9715 |
| *PXO_RS04635* | Lipoprotein | 3.2145 |
| *PXO_RS05155* | glycine rich protein | 3.6984 |
| *PXO_RS10170* | membrane protein | 3.7981 |
| *PXO_RS19085* | L-Ala-D-Glu endopeptidase | 2.9122 |
| *PXO_RS20130* | Small-conductance mechanosensitive channel | 2.5913 |
| *PXO_RS24195* | membrane protein | 2.0107 |
| Signal transduction mechanism (6.82%) | | |
| *PXO_RS06745* | Cyclic di-GMP phosphodiesterase PdeB | 2.1457 |
| *PXO_RS23555* | Two-component system regulatory protein | 2.5661 |
| *PXO_RS25465* | putative signal protein with GGDEF domain | 2.9345 |
| Secondary metabolites biosynthesis, transport and metabolism (34.09%) | | |
| *ligK* | 4-carboxy-4-hydroxy-2-oxoadipate aldolase/oxaloacetate decarboxylase | 2.5361 |
| *cysK* | cysteine synthase A | 2.5894 |
| *PXO_RS02915* | Bacterioferritin-associated ferredoxin | 2.9227 |
| *PXO_RS05600* | Extracellular protease | 2.6875 |
| *PXO_RS07755* | Carboxypeptidase-related protein | 2.5911 |
| *PXO_RS08335* | Chemoreceptor glutamine deamidase CheD | 2.6377 |
| *PXO_RS11220* | Methyl-accepting chemotaxis protein | 3.2487 |
| *PXO_RS13795* | Ferrichrome-iron recepto | 3.5891 |
| *PXO_RS18135* | 4-hydroxy-2-oxovalerate aldolase | 2.0774 |
| *PXO_RS18140* | TonB-dependent outer membrane Receptor | 2.2809 |
| *PXO_RS18145* | Carboxylate—amine ligase | 2.5294 |
| *PXO_RS18150* | Vibrioferrin biosynthesis protein PvsB | 2.8017 |
| *PXO_RS19440* | Sugar transporter | 2.6375 |
| *PXO_RS19445* | TonB-dependent receptor | 2.5016 |
| *PXO_RS22015* | Hemagglutinin | 3.5632 |
| Energy production and conversion (2.27%) | | |
| *hppA* | Pyrophosphatase | 2.7734 |
| Pathogenic-related (6.82%) | | |
| *PXO_RS08465* | Flagellar motor protein MotD | 2.5998 |
| *PXO_RS13190* | TAL effector protein | 2.1961 |
| *PXO_RS14965* | Pilus biogenesis protein | 3.5689 |
| Unknown (25%) | | |
| *PXO_RS04640* | Hypothetical protein | 2.5894 |
| *PXO_RS05080* | Hypothetical protein | 2.7177 |
| *PXO_RS05645* | Hypothetical protein | 2.7767 |
| *PXO_RS21540* | Uncharacterized protein | 2.7415 |
| *PXO_RS11225* | Hypothetical protein | 2.5681 |
| *PXO_RS14880* | Hypothetical protein | 3.5984 |
| *PXO_RS19080* | Hypothetical protein | 2.5566 |
| *PXO_RS21295* | Hypothetical protein | 2.0601 |
| *PXO_RS21410* | Hypothetical protein | 2.6314 |
| *PXO_RS21545* | Hypothetical protein | 3.7254 |
| *PXO_RS22710* | Hypothetical protein | 2.1478 |
| **Down-regulated genes (35)** | | |
| Replication, recombination and repair (14.28%) | | |
| *PXO_RS00600* | Transposase | 0.3853 |
| *PXO_RS00880* | Transposase | 0.3398 |
| *PXO_RS02925* | ISXo5 transposase | 0.4804 |
| *PXO_RS09845* | MutT/nudix family protein | 0.4924 |
| *PXO_RS14390* | DNA ligase-associated DEXH box helicase | 0.4549 |
| Cell wall/membrane/envelope biogenesis (8.57%) | | |
| *PXO_RS07380* | Putative type VI secretion system protein | 0.4179 |
| *PXO_RS19945* | Non-hemolytic phospholipase C | 0.5000 |
| *PXO_RS25700* | Type IV secretion protein Rhs | 0.1071 |
| Secondary metabolites biosynthesis, transport and metabolism (31.43%) | | |
| *bfr* | Bacterioferritin | 0.4384 |
| *PXO_RS04740* | Potassium-transporting ATPase subunit KdpA | 0.4956 |
| *PXO_RS08060* | D-galactonate dehydratase | 0.3525 |
| *PXO_RS08065* | 2-dehydro-3-deoxy-6-phosphogalactonate aldolase | 0.4625 |
| *PXO_RS10475* | Peptidase | 0.3430 |
| *PXO_RS13400* | Methionine synthase | 0.2894 |
| *PXO_RS13405* | Methionine synthase | 0.3124 |
| *PXO_RS13775* | Ferroxidase | 0.4890 |
| *PXO_RS15615* | Vitamin B12 transporter BtuB | 0.4563 |
| *PXO_RS19360* | C4-dicarboxylate transport protein | 0.4937 |
| *PXO_RS21055* | Methionine synthase | 0.4769 |
| Transcription (5.71%) | | |
| *PXO_RS06545* | DNA-binding protein | 0.3489 |
| *PXO_RS13395* | Transcriptional regulator | 0.4512 |
| Pathogenic-related (11.43%) | | |
| *PXO_RS01060* | Glucans biosynthesis protein D (GbpD) | 0.4712 |
| *PXO_RS06380* | Chemotaxis protein CheA | 0.4178 |
| *PXO_RS11830* | Chemotaxis protein | 0.3578 |
| *PXO_RS15905* | Exopolysaccharide xanthan biosynthesis glycosyltransferase GumD | 0.4435 |
| Unknown (28.58%) | | |
| *PXO_RS01615* | Hypothetical protein | 0.4563 |
| *PXO_RS02115* | Hypothetical protein | 0.4789 |
| *PXO_RS03505* | Hypothetical protein | 0.4896 |
| *PXO_RS10195* | Hypothetical protein | 0.3125 |
| *PXO_RS10465* | Hypothetical protein | 0.4609 |
| *PXO_RS16120* | Hypothetical protein | 0.4247 |
| *PXO_RS17710* | Hypothetical protein | 0.4431 |
| *PXO_RS20695* | MEKHLA domain-containing protein | 0.5094 |
| *PXO_RS23565* | Hypothetical protein | 0.3734 |
| *PXO_RS25955* | Hypothetical protein | 0.4681 |
