# Supplementary material for: Contribution of the cold shock protein CspA to virulence in Xanthomonas oryzae pv. oryzae
Source: Mol Plant Pathol. 2018 Nov 16;20(3):382–91. doi: 10.1111/mpp.12763 (PMC6637868; doi:10.1111/mpp.12763)
Supplement: Supplementary file 5 — Table S4 Nineteen putative CspA DNA‐binding genes identified by chromosome immunoprecipitation and high‐throughput sequencing (ChIP‐seq) analysis. [file MPP-20-382-s005.docx]

**Table S4. 19 putative CspA DNA-binding genes identified by ChIP-seq analysis.**

| **Genes** | **Annotation** |
| --- | --- |
| *atpD* | ATP synthase subunit beta |
| *dnaK* | Chaperone protein DnaK |
| *tuf* | Elongation factor Tu |
| *groL* | 60 kDa chaperonin |
| *atpA* | ATP synthase subunit alpha |
| *ftsZ* | Cell division protein FtsZ |
| *PXO_RS11830* | Chemotaxis protein |
| *PXO_RS06155* | Hypothetical protein |
| *trpD* | Anthranilate phosphoribosyltransferase |
| *xseB* | Exodeoxyribonuclease 7 small subunit |
| *rpsE* | 30S ribosomal protein S5 |
| *fusA* | Elongation factor G |
| *PXO_RS14600* | Histidine kinase/response regulator hybrid protein |
| *PXO_RS17505* | Uncharacterized protein |
| *PXO_RS01795* | GTP-binding protein |
| *rpoC* | DNA-directed RNA polymerase subunit beta |
| *PXO_RS01060* | Glucan biosynthesis protein D |
| *PXO_RS00600* | Transposase |
| *PXO_RS02115* | Uncharacterized protein |
